# Factors associated with COVID-19 knowledge among Ghanaians: A national survey

**DOI:** 10.1371/journal.pone.0276381

**Published:** 2022-11-10

**Authors:** Mavis Pearl Kwabla, Juliana Nyasordzi, Gideon Kye-Duodu, Mark Kwame Ananga, Gregory Kofi Amenuvegbe, Joseph Otoo, Dominic Demateh Nuertey, Ebenezer Kofi Mensah, Kwadwo Asante-Afari, Dacosta Aboagye, Joana Ansong, Sally-Ann Ohene

**Affiliations:** 1 Department of Epidemiology and Biostatistics, F.N. Binka School of Public Health, University of Health and Allied Sciences, Ho, Volta Region, Ghana; 2 Department of Nutrition and Dietetics, School of Allied Health Sciences. University of Health and Allied Sciences, Ho, Volta Region, Ghana; 3 Department of Population and Behavioural Sciences, F.N. Binka School of Public Health, University of Health and Allied Sciences, Ho, Volta Region, Ghana; 4 Department of Health Policy Planning and Management, F.N. Binka School of Public Health, University of Health and Allied Sciences, Ho, Volta Region, Ghana; 5 Statistics Department, University of Ghana, Legon, Ghana; 6 Ghana Health Service, Greater Accra Regional Health Directorate, Accra, Ghana; 7 Public Health Reference laboratory, Western and Central Region, Ghana; 8 Ghana Health Service, Health Promotion Division, Headquarters, Accra, Ghana; 9 The WHO, Country office, Accra, Ghana; University of Uyo, NIGERIA

## Abstract

**Background:**

Adequate knowledge about COVID-19 in a population may be relevant in the fight to control its spread among the populace. Thus, the aim of this study was to assess the factors associated with real knowledge of COVID-19 among Ghanaians to promote effective dissemination of appropriate information aimed at containing the spread.

**Methods:**

A cross-sectional online survey and computer assisted telephone interviews (CATI) was conducted among Ghanaians aged 18 years and above across the 260 districts of Ghana. The survey assessed the level of knowledge of COVID-19 and its associated factors and compared differences between perceived and real knowledge. One district health promotion officer per district was trained for the data collection. Participants were recruited via use of phone directories of both organized and non-organized local district groups. Phone calls were made to randomly selected phone contacts to schedule options for participation in the study. We used multivariable logistic regression to investigate the associated factors of COVID-19 knowledge among respondents.

**Results:**

Of the 2,721 participants who completed the survey, the majority (99.3%) were aware of the existence of the COVID-19 outbreak, had good knowledge on infection prevention (87.0%) and rated their knowledge about COVID-19 as good (81.7%). Factors associated with COVID-19 knowledge were: age ≥56 years (aOR = 0.5; CI: 0.3–0.8; p = 0.002), tertiary education (aOR = 1.8; CI: 1.2–2.6; p = 0.003), residing in Greater Accra region (aOR = 2.0; CI: 1.1–3.6; p = 0.019), not infected with the novel coronavirus (aOR = 1.5; Cl: 1.0–2.1; p = 0.045), knowing an infected person (aOR = 3.5; CI = 1.5–7.9; p = 0.003), good practice of effective preventive measures (aOR = 1.2: Cl: 1.1–1.5: 0.008), not misinformed (aOR = 0.7; Cl: 0.5–0.9; 0.015), and perceiving spreading speed of the virus as slow (aOR = 0.7; Cl: 0.5–0.9; 0.007).

**Conclusion:**

The study found good knowledge regarding COVID-19, control measures, and preventive strategies. The Ghana Health Service should continuously provide accurate information to educate the media and citizens to prevent misinformation, which is vital in stopping the spread of the COVID-19 virus.

## Introduction

The devastating impacts of the coronavirus pandemic (COVID-19) on the global health systems, economies and general human life have been felt in every continent since its first outbreak in 2019 [1–3]. WHO declared the COVID-19 epidemic as a public health emergency of international concern on January 30, 2020 [1]. Globally there has been 611,552,387 confirmed COVID-19 cases with 6,525,415 deaths as at September 16, 2022. The first case in Africa was reported in Egypt on February 14, 2020 [4]. Since then 55 countries in Africa currently have reported 12,630,940 confirmed cases and 257,523 confirmed deaths as at September 16, 2022 at 7:25pm [5]. Ghana reported its first two imported cases on March 12 2020 [6]. As of September 16 2022, Ghana had recorded 168,616 confirmed cases with 1,459 deaths [5].

COVID-19 is an emerging disease with significant threats to public health. Some of the signs and symptoms of the disease are cough, shortness of breath, fatigue, fever, myalgia and dyspnea [7]. Interventions such as travel restrictions, partial lock down in some parts of Ghana were instituted after the confirmation of the disease in the country. Additionally, the wearing of facemask, hand washing, use of sanitizers and social distancing [8] were enforced by the Ministry of Health and the government in a bid to curb the spread of the disease. However, adherence to government protocols seems to be poor and the spread continues to increase. Some studies on knowledge and attitudes during epidemics indicate the populace tend to be reluctant in adopting instituted control measures [9, 10], because they caused discomfort.

It has been indicated that the majority of people infected with COVID -19 would be asymptomatic [11], however the rapid spread of the disease through symptomatic or asymptomatic infected individuals, [12] warrants identification of behavioral responses of the populace to mitigate the spread of the disease [13]. Studies show that knowledge of the populace is essential in addressing pandemics [14–16]. A study in Hubei assessing knowledge among other factors about COVID-19 reported that reaction of people towards government protocols to contain the disease are strongly related to the level of knowledge about COVID-19 [17]. Also the higher the level of information, and education, the more likely individuals would put up a positive attitude towards COVID-19 preventive practices [17]. This suggests that inadequate or lack of knowledge about COVID-19 could be a mediator that can increase transmission of the virus [11].

COVID-19 is a new disease, with emerging discoveries every now and then, sometimes accompanied with misinformation [15, 16]. Misconceptions and inappropriate knowledge about COVID-19 have been reported in Ghana [16]. As a result, to effectively manage the disease, it is important to assess the knowledge level, risk perception of the populace on a regular basis. This will aid in the dissemination of discoveries about the mode of spread and preventive measures, thus helping in the fight against the disease in Ghana [16]. Adequate knowledge of COVID-19 may enable the populace to adhere to instituted government protocols and other preventive measures [16].

As at the time of this study, only a few studies on COVID-19 related knowledge, attitude and practices had been conducted among a section of Ghanaians [18–20] but, a study covering a wide section of the populace in all the regions of Ghana was not available. However, the larger the number of study participants, the higher the likelihood of the study validity and generalizability [12]. Thus, it is essential to present data among a relatively large number of Ghanaians to monitor variables critical for behaviour change in the population. This study seeks to assess knowledge of COVID-19 among Ghanaians in all the regions of Ghana to help identify gaps, promote effective communication and timely dissemination of appropriate information aimed at reducing further transmission of COVID-19 to help contain the pandemic.

## Methods

### Study area

**Fig 1:**
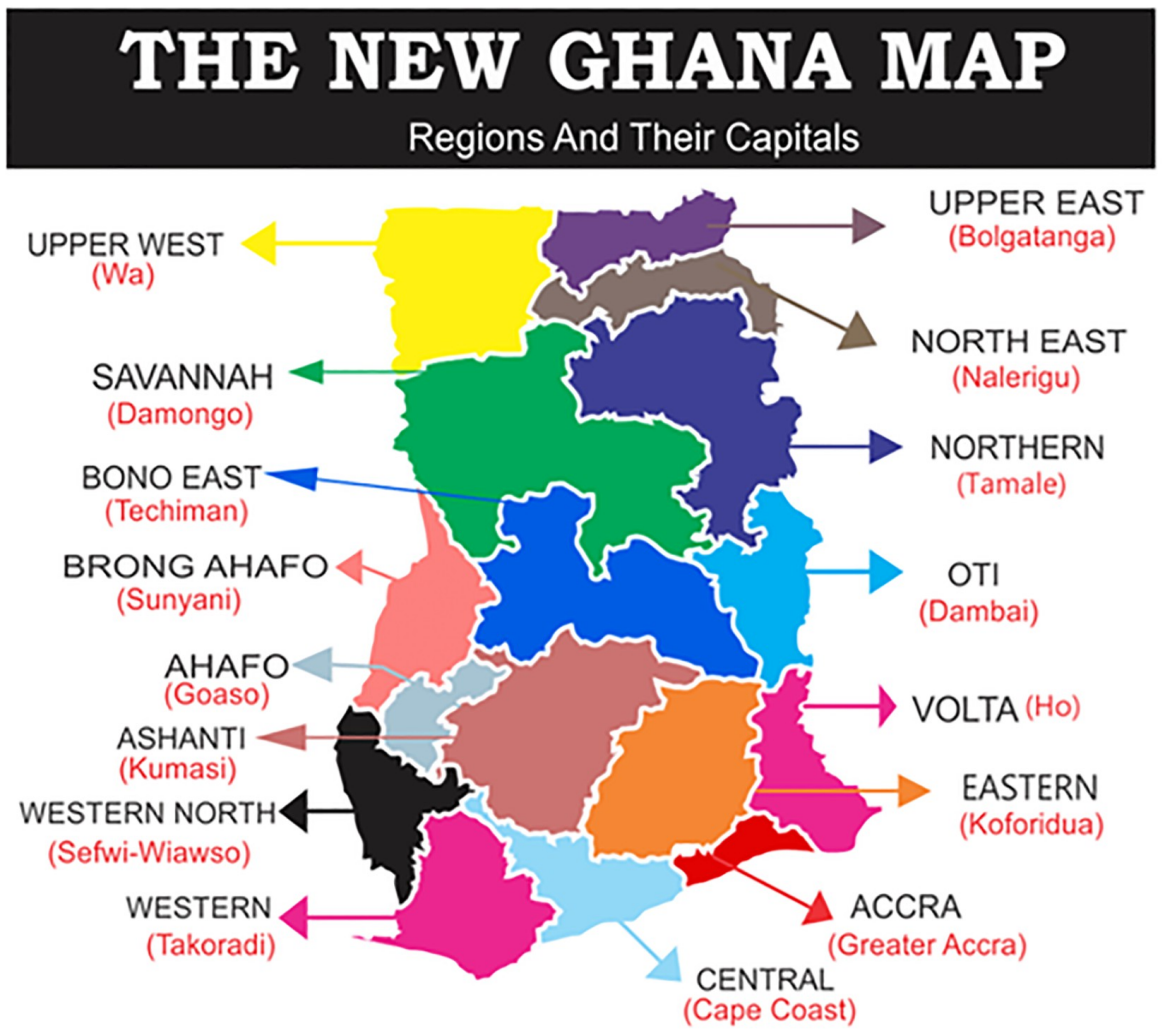
Map of Ghana with the regions and regional capital towns in brackets.

This study was conducted nationwide across all 260 districts from all the 16 regions of Ghana (Fig 1: Map of Ghana).

### Data collection procedure/recruitment of participants

Between August and December 2020, we conducted an online survey and computer assisted telephone interviews (CATI) using a cross-sectional design among Ghanaians aged 18 years and above to assess the level of knowledge of COVID-19 and its associated factors. District health promotion officers served as data collectors. In each of the 260 districts, one district health promotion officer was selected and trained for the data collection. To minimize spread of the virus via face-to-face contacts, we employed online platforms such as use of mobile phones and computers for the data collection. Participants were recruited via use of phone directories of local district groups including both organized (e.g. district health facilities) and non-organized groups (e.g. transport unions). This was followed by placing phone calls to randomly selected phone contacts to schedule options for participation and time; the content of the questionnaire was not made known to participants at this point and they were instructed not to start the survey until they have enough time to complete it at a go. Potential participants with smart phones who opted to take the survey online by themselves were sent the questionnaire while those with or without smart phones who opted for assistance in taking the survey were interviewed via the phone by the health promotion officers. The interviews via phone calls was necessary to create a balance of both elderly and young population in the study and as well cater for those who do not have any formal education to take the interviews in their local spoken languages.

The questionnaire was generated using Google forms. A link to the questionnaire was shared with prospective respondents who opted to take the survey by themselves via WhatsApp or email and was either filled in, on their mobile phones or computers. Those who indicated they could not read or write, but consented to participate in the study, had an appointment scheduled for interviewing via the mobile phone. The health promotion officer called the participants on the scheduled time and the questionnaire is read to them in a dialect mutually understood by the health promotion officer (resident in the district) and the participant. The health promotion officer enters the responses to the questions directly into the Google platform either using a mobile phone or computer. The health promotion officers were provided with call credits for this exercise. To limit the responses of participants, they were required to sign in with a Google account, with each limited to a single response. Completion of a single questionnaire took approximately 30 minutes.

### Study sample and sampling process

The study employed a cross-sectional design. Proportionate sampling technique that is representative of the 260 districts of Ghana was employed, that is sample selected in each region and district was proportional to the population size. We used the Cochran formula $n=\frac{Z^{2}\;pq}{e^{2}}$ (p:proportion of real knowledge of COVID-19 unknown hence 50% was used; z: z value based on 95% confidence level and e: margin of error of 2% used) [21] to arrive at the estimated sample size of 2641 and used proportionate sampling to estimate the number of respondents to be recruited from each region and district. To randomly select estimated sample for each district, we exported the data on phone directories to STATA MP 16.0 software and the desired number estimated was selected. However, to make up for non-response in some regions, we increased the number of respondents for the regions with high COVID-19 prevalence (Greater Accra, Volta, Eastern and Central regions). This practice is similar to what was done in a national survey in the Netherlands [22] and hence respondents for these regions were more than the estimated sample size.

### Inclusion and exclusion criteria

Ghanaians 18 years and above residing in the districts 12 months prior to the study were included in the study. All those who meet the above criteria but for the purpose of mental or physical health or refuse to give consent were excluded.

### Study variables and data collection tools

We used an adapted WHO standardized questionnaire [23] to collect data on participant’s sociodemographic characteristics as well as on knowledge related factors. The questionnaire can be found here: https://hdl.handle.net/20.500.12034/2392.

The outcome was real knowledge of COVID-19 measured as composite of 22 out of 31 knowledge variables selected using principal component analysis (PCA) for use in assessing knowledge of participants. The average real knowledge score was 17.7 (CI: 17.54–17.76) and was categorized as poor (knowledge score of below 18) and good (knowledge score of 18 and above). Perceive knowledge used the question; how would you rate your level of knowledge on the novel coronavirus and was rated on a scale ranging from 1 (very little knowledge) to 7 (very much knowledge) but were re-categorized as poor knowledge (scale 1–4) and good knowledge (scale 5–7). Other independent variables assessed were presence of chronic diseases, being infected or knowing someone infected with the virus, a feeling of preparedness and self-efficacy to avoid an infection with the coronavirus to measure protective behaviours of participants including how to protect one’s self from the virus, following recommendations to prevent spread of the virus and whether participants think it is difficult or easy to avoid getting infected. We also assessed effective preventive measures and its uptake, misinformation about protective measures (percent of wrong protective measures identified as effective), perceiving the pandemic as a media hype and whether knowledge was related to certain sources of information.

### Ethical issues

Ethical approval for the study was granted by the Ghana Health Service Ethics Review Committee (reference number GHS-ERC 004/06/20). This was done according to the accepted guidelines of the Committee on Ethics in Human Experimentation and the International Council for Harmonization (ICH)/Good Clinical Practice (GCP). This study was carried out in collaboration with the Health Promotion Division of the Ghana Health Service which provided an institutional support letter for the field activities. All participants were given adequate information on the study protocol and participation was voluntary. Being an online survey and computer assisted telephone interviews, informed consent was in the form of participants ticking a box to indicate their agreement to take part in the study and this box has to be ticked before having access to the questionnaire. Confidentiality of data was assured for all information provided.

### Statistical analysis

The data used for this analysis was part of a larger study data looking at behavioural insights for COVID-19 in Ghana. We subjected the dataset for the study to both descriptive and inferential statistical analyses. For continuous variables, we performed summary statistics and checked for normality using S-Wilk test and reported the mean and standard deviation; chi-square test was used for all categorical variables. We used principal component analysis (PCA) to identify the variables used in measuring knowledge. For inferential statistics, we used multivariable logistic regression analyses to investigate the associated factors of COVID-19 knowledge among the study participants. We used forward selection to select significant independent variables from the bivariate model into the multivariable model. After adjusting for confounding, all independent variables with a p-value of <0.05 were considered statistically significant. We performed all the analyses using STATA MP 16.0 software.

## Results

### Demographic characteristics of participants, Ghana, 2020

Of the 2,721 participants who completed the survey, 1,490 (54.2%) were males, 1,648 (60.6%) had tertiary or university level education, 1,395 (51.3%) are engaged in formal employment, 1.288 (51.9%) were urban residents and were aged between 18 and 89 years old (Table 1).

**Table 1:** Demographic characteristics of participants, Ghana, 2020.

Characteristics	Category	Frequency	Percentage (%)
**Gender (n = 2721)**			
	Male	1231	45.2
	Female	1490	54.7
**Age (years) (n = 2715)**		* ***M = 34***	^∞^ ***SD = ±11*.*2***
	18–25	594	21.9
	26–35	1238	45.6
	36–45	503	18.5
	46–55	210	7.7
	56+	170	6.3
**Education level (n = 2721)**			
	No formal education	274	10.1
	Primary/Middle/Secondary	799	29.4
	Tertiary/University	1648	60.6
**Occupation (n = 2721)**			
	Unemployed	425	15.6
	Informal	901	33.1
	Formal	1395	51.3
**Regional responses (n = 2721)**			
	Ahafo	67	2.5
	Ashanti	367	13.5
	Bono East	134	4.9
	Brong Ahafo	86	3.2
	Central	290	10.7
	Eastern	369	13.6
	Greater Accra	583	21.4
	North East	6	0.2
	Northern	128	4.7
	Oti	43	1.6
	Savannah	102	3.8
	Upper East	57	2.1
	Upper West	97	3.6
	Volta	257	9.5
	Western	52	1.9
	Western North	83	3.05
**Residence (n = 2480)**			
	Rural	1192	48.1
	Urban	1288	51.9

*M = Mean;

^**∞**^***SD*** = Standard Deviation

Of the 2,721 participants who completed the survey, 2,702 (99.3%) were aware of the novel coronavirus pandemic (Table 2). Compared to males, a higher proportion of females (54.7%) were aware of the existence of COVID-19 pandemic. Knowledge of the common symptoms of the coronavirus such as fever, cough and shortness of breath were 95.6%, 97.7% and 96.3% respectively and on person-to-person transmission was 95.8%. On management of COVID-19, 2068 (76.0%) believed there was no drug for treatment or vaccine at the time of the study. Correct knowledge of incubation period of up to 14 days was 80.6%. More than half of participants, 1654 (60.8) believed recovery from infection with the virus does not confer immunity to the disease while a few 575 (21.1%) believe it does confer immunity. While the majority (97.0%) believe people aged 60 years and older are at a higher risk of getting COVID-19, two-thirds (75.9%) also believe children aged 1–5 years are part of the risk groups (Table 2).

**Table 2:** Perceive and real knowledge of COVID-19 among participants.

Variables	Frequency	Percentage (%)
**Knowledge level on awareness of the novel coronavirus**		
Yes	2702	99.3
No	19	0.7
**The following are symptoms of the novel coronavirus #**		
Fever	2602	95.6
Cough	2659	97.7
Shortness of breath	2620	96.3
Sore throat	2493	91.6
Runny or stuffy nose	2463	90.5
Muscle or body aches	2038	74.9
Headaches	2460	90.4
Fatigue (tiredness)	2269	83.4
Diarrhea	1497	55.0
**Knowledge on transmission of COVID-19**		
The novel coronavirus is transmissible from person to person	2592	95.8
The novel coronavirus is transmitted by animals to humans only	66	2.4
The novel coronavirus is not transmissible	9	0.3
Don’t know *	39	1.4
COVID-19 is transmissible via droplets through coughing, sneezing or intimate contact	2643	97.1
The novel coronavirus is transmissible via the fecal-oral route	28	1.0
Black races and those in tropical countries are less susceptible to the novel coronavirus	22	0.8
Don’t know	28	1.0
**Knowledge on management of COVID-19)**		
There is a drug to treat the novel coronavirus	306	11.3
There is a vaccine for the novel coronavirus	90	3.3
There is both a drug for the treatment and a vaccine for the novel coronavirus	71	2.6
There is currently no drug treatment or vaccine for the novel coronavirus	2068	76.0
Don’t know	186	6.8
**Knowledge about incubation period** **What is the incubation period**		
Up to 3 days	89	3.3
Up to 7 days	211	7.8
Up to 14 days	2193	80.6
Don’t know	228	8.4
**Which of these are correct about the COVID-19 disease?**		
Recovery from coronavirus confers immunity to COVID-19	575	21.1
Recovery from coronavirus does not necessarily confers immunity to COVID-19	1654	60.8
Don’t know	492	18.1
**Knowledge on groups at risk of severe illness related to the novel coronavirus**	**At risk**	**Not at risk**
People aged 60 years or older	2638 (96.95)	83 (3.1)
Pregnant women	2389 (87.80)	332 (12.2)
Infants	2117 (77.80)	604 (22.2)
Children aged 1–5 years	2066 (75.93)	655 (24.1)
People who have serious chronic heart disease	2539 (93.31)	182 (6.7)
People who have serious chronic diabetes	2524 (92.76)	197 (7.2)
People who have serious lung disease	2549 (93.68)	172 (6.3)
People who have asthma	2539 (93.31)	182 (6.7)
Overall real knowledge	18±3	
Poor	929	34.1
Good	1792	65.9
**Perceived knowledge**		
***Rating knowledge level on the novel coronavirus***	6±1	
Poor (1–4)	491	18.0
Good (5–7)	2230	82.0
***Knowledge level on how to prevent spread of the novel coronavirus***	6±1	
Poor (1–4)	355	13.1
Good (5–7)	2366	86.9

Over two-third of participants 2439 (86.6%) who indicated they know how to protect themselves from the virus (perceive self-efficacy) also follow the recommendations to prevent spread of the virus 2437 (89.6%) (Table 3). While majority 1998 (73.4%) are indifferent regarding how to avoid infection with the virus, a few however indicated it is extremely difficult 333 (12.2%) and 390 (14.3%) said it is extremely easy. More than half 1796 (66.0%) of the study population were indifferent about receiving the coronavirus vaccine when made available (Table 3). Questions on preparedness and perceive self-efficacy as well as rating ones knowledge level on how to prevent the spread of the novel coronavirus are based on a Likert scale from 1–7.

**Table 3:** Participants feelings about preparedness and self-efficacy to avoid an infection with the coronavirus.

Preparedness and perceived self-efficacy			
***I know how to protect myself from coronavirus***	***Mean = 6*.*05*** ***Frequency***		^∞^ ***SD = ±1*.*2*** ***Percentage***
Not at all (1–3)	102		3.8
Indifferent (4)	2439		89.6
Very much so (5–7)	180		6.6
***I follow the recommendations from authorities in my country to prevent spread of novel coronavirus***	***Mean = 6*.*07***		***SD = ±1*.*2***
Not at all (1–3)	99		3.6
Indifferent (4)	185		6.8
Very much so (5–7)	2437		89.6
***For me avoiding an infection with the novel coronavirus in the current situation is…***	***Mean = 5*.*33***		***SD = ±1*.*6***
Extremely difficult (1–3)	333		12.2
Indifferent (4)	1998		73.4
Extremely easy (5–7)	390		14.3
**Willingness to take vaccine (policies)**			
**If a vaccine becomes available and is recommended for me, I would get it.**			
Strongly disagree	588	21.6	
Indifferent	1796	66.0	
Strongly agree	337	12.4	

^∞^SD = Standard deviation

### Knowledge and uptake of protective measures

**Fig 2:**
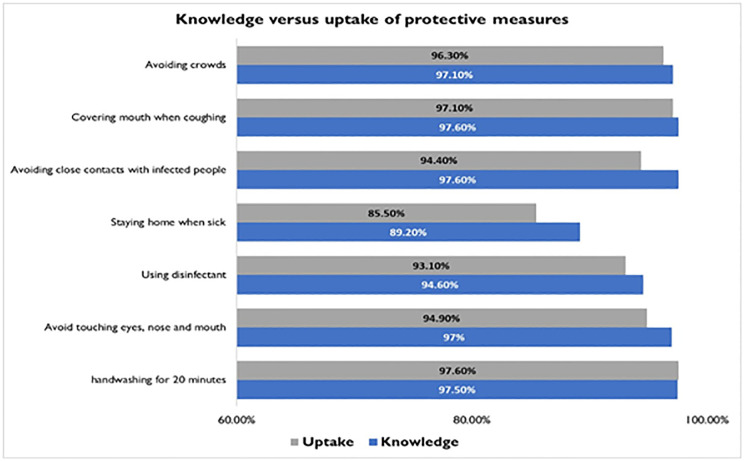
Knowledge of protective measures versus uptake of protective measures.

We assessed participants who rated protected measures as effective and compared it with the actual use of those protective measures. We found that almost equal proportion of participants with knowledge of effective protective measures also practice the measures (Fig 2).

**Fig 3:**
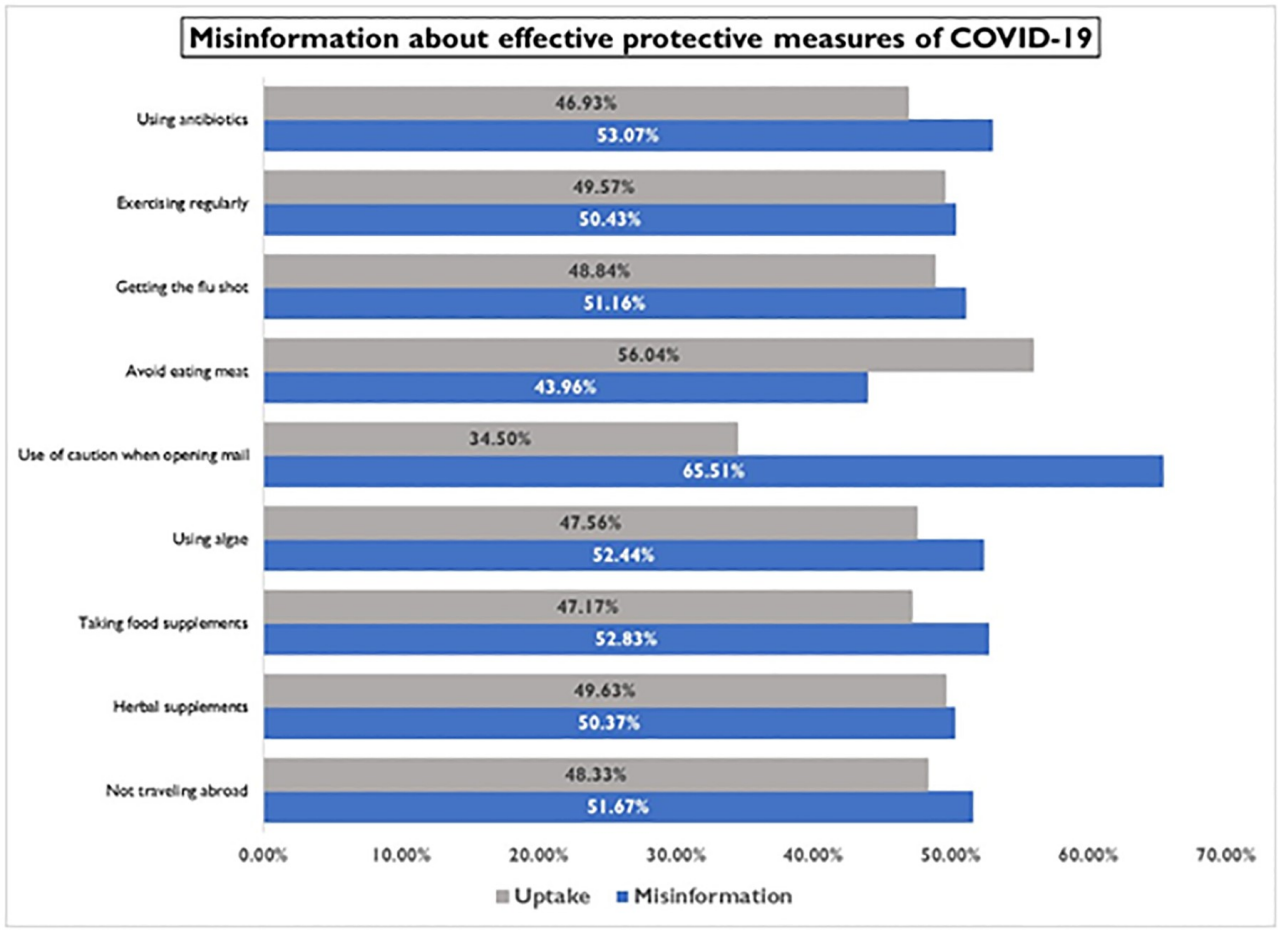
Misinformation about effective protective measures.

We assessed the proportion of non-effective preventive measures that were wrongly identified as effective. Non-effective preventive measures identified by participants as effective were refusing to travel abroad (81), taking food supplements (67), taking caution when opening a mail (38), drinking ginger water (57) and using antibiotics (38) (Fig 3).

### Real versus perceived knowledge of COVID-19 among participants

**Fig 4:**
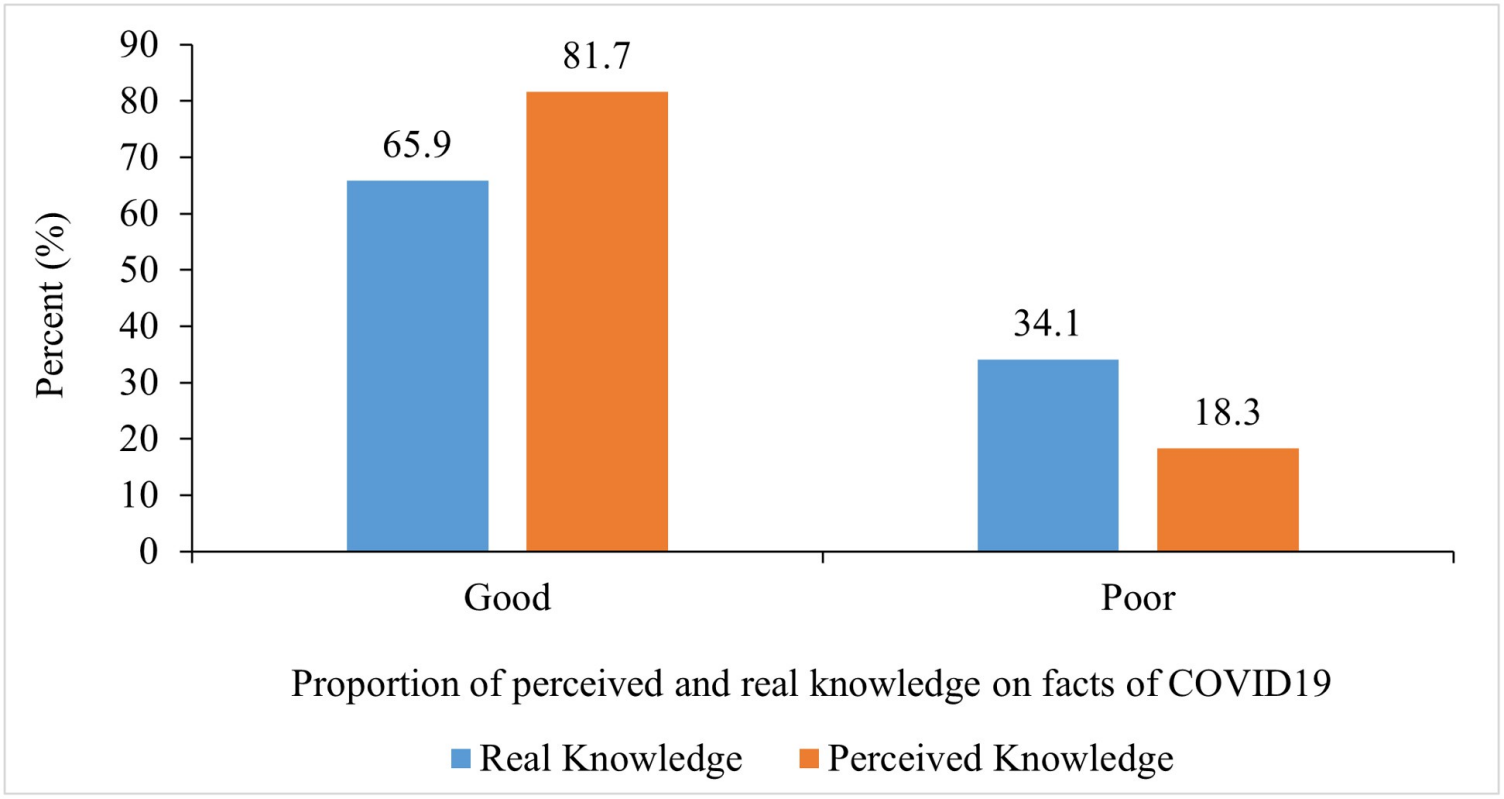
Perceived knowledge against real knowledge on facts of COVID-19.

Overall assessment of perceived and real knowledge on the facts of COVID-19 indicated that a higher proportion of participants (81.7%) rated their knowledge as good but a test of understanding with questions on basic facts of the virus showed only 65.9% had actual good knowledge (Fig 4).

### Trust/Confidence in information

**Fig 5:**
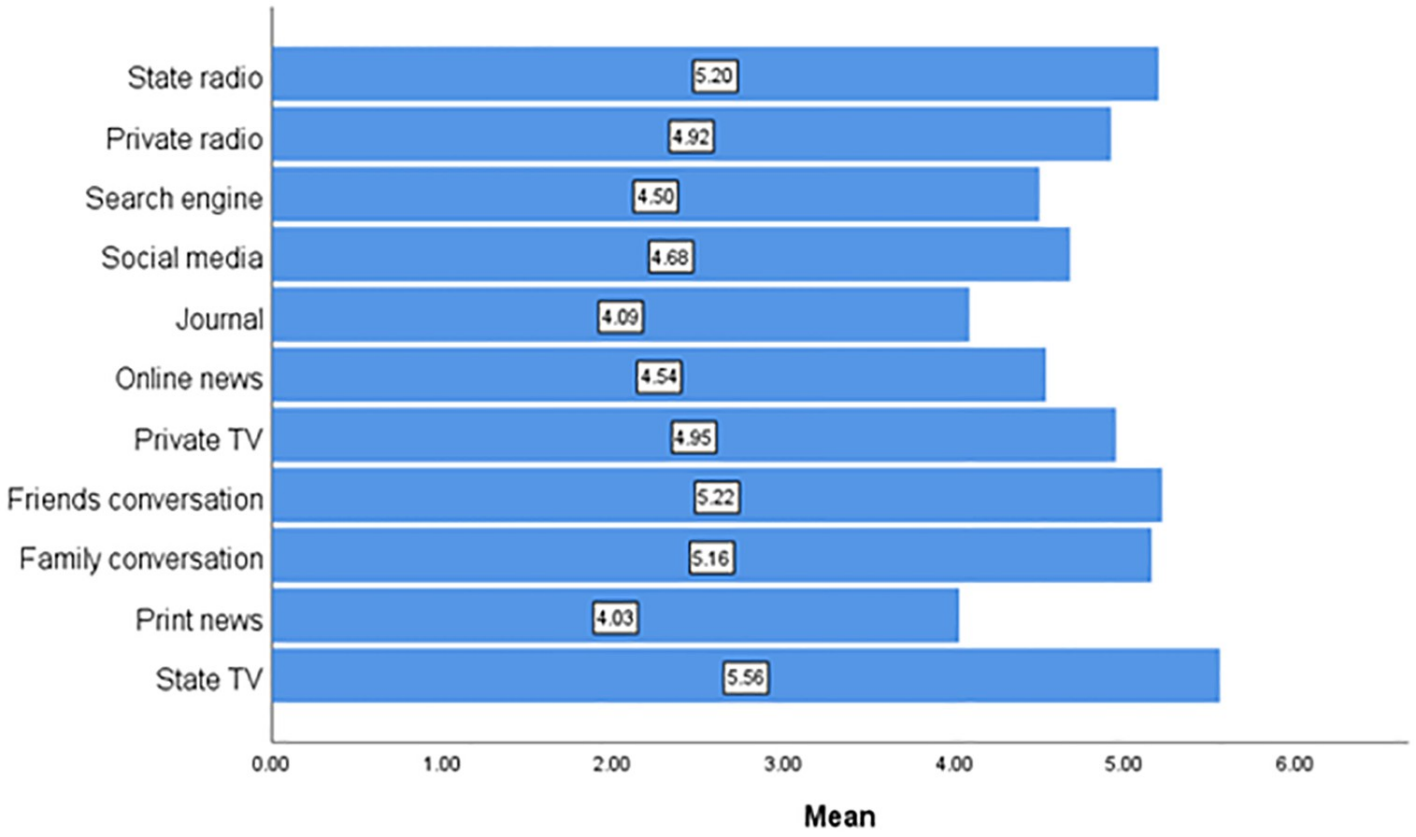
Trust/confidence in media.

Participants were asked about how much confidence they have in individuals and/or organizations that were handling the novel coronavirus and responses were assessed on 7-point scales from very low confidence to very high confidence. The mean highest level of trust were state TV (5.6), state radio (5.2) and information received through conversation with friends (5.2) (Fig 5).

### Factors associated with COVID-19 knowledge among participants

The study found nine variables to be significantly associated with COVID-19 knowledge among Ghanaians (Table 4). Increasing age of 56 years and above decreases the odds of having a good knowledge by 49% compared to younger age groups (OR = 0.51;CI = 0.3–0.8; p = 0.002). Participants who had attained a tertiary level of education were 1.8 times more likely to have good knowledge of COVID-19 than those with informal education (aOR = 1.8; CI: 1.2–2.6; p = 0.003). Residing in the Greater Accra region was associated with 2.0 times the odds of having knowledge about COVID-19 (aOR = 2.0; CI = 1.1–3.6; p = 0.019) compared to other regions. On the other hand, respondents who reside in the Upper East region were 0.4 times less likely to have good knowledge of COVID -19 than those in other regions (aOR = 0.4; CI = 0.2–1.0; p = 0.038). Participants who were not infected with the novel coronavirus during the study period were 1.5 times more likely to have had good knowledge of COVID-19 than those who were not sure of their COVID-19 status (aOR = 1.5; Cl: 1.0–2.1; p = 0.045). Similarly, respondents who knew someone confirmed as a COVID-19 case were 1.9 times more likely to have good knowledge of COVID-19 (aOR = 1.9; CI = 1.2–2.8; p = 0.003) compared to those who do not know an infected person.

**Table 4:** Factors associated with COVID-19 knowledge among Ghanaians.

	Knowledge level		Univariate regression		Multivariate regression	
	Poor	Good	OR (95%CI)	P-value	AOR (95%CI)	P-value
	n (%)	n (%)				
**Age (years)**						
18–25	217 (23.4)	377 (21.1)	1 (Reference)		1 (Reference)	
26–35	369 (39.8)	869 (48.6)	1.4 (1.1–1.7)	0.004*	1.1 (0.8–1.4)	0.575
36–45	163 (17.6)	340 (19.0)	1.2 (0.9–1.5)	0.153	1.3 (0.9–1.8)	0.107
46–55	82 (8.8)	128 (7.2)	0.9 (0.7–1.2)	0.517	1.15 (0.8–1.7)	0.510
56+	97 (10.5)	73 (4.1)	0.4 (0.3–0.6)	0.000 *	0.51 (0.3–0.8)	0.002*
**Education level**						
No school	139 (15.0)	135 (7.5)	1 (Reference)		1 (Reference)	
Primary/Middle/Secondary	359 (38.6)	440 (24.6)	1.3 (1.0–1.7)	0.097	1.0 (0.7–1.3)	0.792
Tertiary/University	431 (46.4)	1217 (67.9)	2.9 (2.2–3.8)	0.000 *	1.8 (1.2–2.6)	0.003*
**Region**						
Ahafo	27 (2.9)	40 (2.2)	1 (Reference)		1 (Reference)	
Ashanti	127 (13.7)	240 (13.4)	1.3 (0.8–2.2)	0.371	1.0 (0.6–1.8)	0.957
Bono East	42 (4.5)	92 (5.1)	1.5 (0.8–2.7)	0.209	1.1 (0.6–2.2)	0.796
Brong Ahafo	36 (3.9)	50 (2.8)	0.9 (0.5–1.8)	0.846	0.9 (0.4–1.8)	0.724
Central	96 (10.3)	194 (10.8)	1.4 (0.8–2.4)	0.265	1.0 (0.6–1.9)	0.902
Eastern	102 (11.0)	267 (14.9)	1.8 (1.0–3.0)	0.038 *	1.6 (0.9–2.9)	0.143
Greater Accra	166 (17.9)	417 (23.3)	1.7 (1.0–2.9)	0.047 *	2.0 (1.1–3.6)	0.019*
North East	3 (0.3)	3 (0.2)	0.7 (0.1–3.6)	0.645	0.7 (0.1–5.3)	0.713
Northern	51 (5.5)	77 (4.3)	1.0 (0.6–1.9)	0.951	0.8 (0.4–1.5)	0.415
Oti	18 (1.9)	25 (1.4)	0.9 (0.4–2.0)	0.871	1.0 (0.4–2.3)	0.982
Savannah	42 (4.5)	60 (3.4)	1.0 (0.5–1.8)	0.910	1.1 (0.5–2.2)	0.855
Upper East	33 (3.6)	24 (1.3)	0.5 (0.2–1.0)	0.052 *	0.4 (0.2–1.0)	0.038*
Upper West	38 (4.1)	59 (3.3)	1.1 (0.6–2.0)	0.885	0.8 (0.4–1.6)	0.555
Volta	111 (12.0)	146 (8.2)	0.9 (0.5–1.5)	0.670	0.8 (0.4–1.4)	0.403
Western	12 (1.3)	40 (2.2)	2.3 (1.0–5.1)	0.049 *	1.2 (0.5–2.9)	0.689
Western North	25 (2.7)	58 (3.2)	1.6 (0.8–3.1)	0.194	1.8 (0.9–3.9)	0.112
**Infected with the novel coronavirus**						
Yes, confirmed	41 (4.4)	88 (4.9)	1.8 (1.1–2.9)	0.014 *	2.0 (1.1–1.1)	0.021*
Yes, not confirmed	10 (1.1)	44 (2.5)	3.7 (1.8–7.8)	0.001 *	3.47 (1.5–7.9)	0.003*
No	790 (85.0)	1555 (86.8)	1.7 (1.2–2.2)	0.001 *	1.46 (1.01–2.1)	0.045*
Don’t know	88 (9.5)	105 (5.9)	1 (Reference)		1 (Reference)	
**Know someone who is infected**						
Yes, confirmed	116 (12.5)	375 (20.9)	2.8 (2.0–3.9)	0.000 *	1.9 (1.2–2.8)	0.003*
Yes, not confirmed	31 (3.3)	56 (3.1)	1.6 (0.9–2.6)	0.091	1.1 (0.6–2.1)	0.682
No	673 (72.4)	1234 (68.9)	1.6 (1.2–2.1)	0.001*	1.4 (1.0–1.9)	0.073
Don’t know	109 (11.7)	127 (7.1)	1 (Reference)		1 (Reference)	
**Knowledge of effective preventive measures**						
Poor	522 (56.2)	811 (45.3)	1 (Reference)		1 (Reference)	
Good	407 (43.8)	981 (54.7)	1.6 (1.3–1.8)	0.000*	1.7 (1.2–2.4)	0.004*
**Practice or uptake of effective preventive measures**						
Poor	425 (45.8)	725 (40.5)	1 (Reference)		1 (Reference)	
Good	504 (54.3)	1067 (59.5)	1.2 (1.1–1.5)	0.008*	0.9 (0.7–1.2)	0.469
**Misinformation about preventive measures**						
Misinformed	503 (54.1)	897 (50.1)	1 (Reference)			
Not misinformed	426 (45.9)	895 (49.9)	1.2 (1.0–1.4)	0.043*	0.7 (0.5–0.9)	0.015*
**Perceived spreading speed**						
Spreading slowly	193 (20.8)	226 (12.6)	0.8 (0.6–1.0)	0.020*	0.7 (0.5–0.9)	0.007*
Moderate	269 (29.0)	422 (23.6)	1 (Reference)		1 (Reference)	
Spreading faster	467 (50.3)	1144 (63.8)	1.6 (1.3–1.9)	0.000*	1.2 (1.0–1.5)	0.098
**Trust in institutions**			1.2 (1.1–1.2)	0.000*	1.1 (1.1–1.2)	0.000*

Those who had good knowledge of effective preventive measures were 1.7 times more likely to have an overall good knowledge of COVID-19 than those who had poor knowledge (aOR = 1.7; Cl: 1.2–2.4; 0.004). Good uptake of effective preventive measures was associated with a 10% decrease in odds of having good knowledge of COVID-19 (aOR = 0.9: Cl: 0.7–1.2: 0.469). Respondents who were not misinformed were 0.7 times less likely to have good knowledge of COVID-19 than those who were misinformed (aOR = 0.7; Cl: 0.5–0.9; 0.015). Respondents who perceived the spread of the COVID-19 virus as slow were 30% less likely to have good knowledge of COVID-19 compared to those who perceived it as moderate (aOR = 0.7; Cl: 0.5–0.9; 0.007).

We did not find any difference between those who trusted the instituted protocols for prevention of the virus and those who did not have trust. However, trust in the instituted prevention protocols was significantly associated with good knowledge.

## Discussion

With the aim of directing communication and the dissemination of important information, our study evaluated Ghanaians knowledge of COVID-19. Our findings indicate that most Ghanaians (99.3) were aware of the COVID-19 outbreak, had a strong understanding of infection prevention (87.0%), and thought their knowledge of COVID-19 was good (81.7%). Additionally, we discovered that those 56 years of age and older had lower knowledge of COVID-19 (aOR = 0.5; CI: 0.3–0.8; p = 0.002), but knowledge increased with tertiary education (aOR = 1.8; CI: 1.2–2.6; p = 0.003), living in the Greater Accra region (aOR = 2.0; CI: 1.1–3.6; p = 0.019) and not having been infected with the novel coronavirus (aOR = 1.5; Cl: 1.0–2.1; p = 0.045).

The findings of this study with regards to decreased knowledge of COVD-19 with increased participant’s age differs from results in studies conducted in Uganda [24] and Saudi Arabia [25, 26] which reported increased knowledge towards COVID-19 with increased age. In a study in Bangladesh, the older respondents, specifically retirees, were more knowledgeable on COVID-19 than the younger participants [27]. Our result is similar to findings from a study conducted in Ethiopia which reported that participants in the ≥65 years age group were 2.72 times more likely to have inadequate knowledge of COVID-19 as compared with the 8–35 years age group (AOR = 2.27, 95% CI 1.45 to 5.11) [28]. Also a study conducted in Egypt among 559 adult Egyptians, reported that knowledge towards COVID-19 was significantly lower among older age respondents [29]. Although the main reason for the observed decreased knowledge towards COVID-19 among the elderly is not precisely known, it is possible this could be due to difficulty in hearing, impaired visual ability and loss of cognition associated with ageing in older people, which might lead to their inability to listen to the media providing education on COVID-19 and to search for information compared to younger people. The case is different from the study conducted in Vietnam, where no association was found between knowledge of COVID-19 and the age groups of the respondents [30]. However, this study suggests that more prominence should be given to targeting older people to improve their knowledge of the COVID-19 pandemic since they are highly at risk of COVID-19 infections due to decreased immunity.

In terms of our finding that respondents who had attained a tertiary level of education were more likely to have good knowledge of COVID-19 than those with informal education, other studies conducted in Ethiopia [31, 32], Iran [33], and Saudi Arabia [12, 26] found poor knowledge of COVID-19 to be significantly associated with respondents with informal education, which is consistent with the present study. Also, it has been indicated in a study in Ethiopia that respondents who were unable to read and write were 60% times more likely to have inadequate knowledge compared with those who attained high-level education (AOR = 1.60, 95%CI 1.02 to 2.51) [28]. The probable reason could be that higher education is linked to higher knowledge and better understanding. It could also be possible that educated people are likely to search for information regarding the COVID-19 pandemic, control measures and preventive strategies since they are exposed to the internet and curious about currents happening in the world. On the contrary, a study conducted in Uganda found no association between knowledge of COVID-19 and educational level [24].

Some studies have also reported differences in knowledge based on participant’s residential area. For instance, while some studies report that urban dwellers are more knowledgeable about COVID-19 than rural counterparts [28], some report no association at all; a study conducted in Saudi Arabia found no association between COVID-19 knowledge and residential area [12]. In our study, residents of the Greater Accra region were more likely to have good knowledge of COVID-19 compared to residents of other regions. The possible relationship is that Accra is the capital city of Ghana, and has a number of media stations, both radio and print media, churning out information about the pandemic to educate the populace. Thus residents in this region have access to different sources of media to acquire information concerning COVID-19. It is generally known that a more educated populace about any type of disease will generally comply better with the preventive and treatment measures issued [34]. The government also had a series of engagement through media broadcast from this region and telecasted to other regions as well, all in a bid to provide education on COVID-19 and to protect citizens.

With our observation that respondents who were not infected with the novel coronavirus were more likely to have good knowledge of COVID-19 than their infected counterparts, it is possible that their adequate knowledge of COVID-19 infections led to non-infection. They are likely to apply their knowledge to observe COVID-19 measures, control and preventions provided to nations by the World Health and Organization (WHO) and the Centers for Disease Control (CDC). In addition, our study found that those who knew someone infected with the COVID-19 virus were more likely to have good knowledge of COVID-19 than those who were not sure. The probable clarification could be that those who knew infected people took precautions to protect themselves by acquiring information about the virus, control measures, and preventive strategies, which might account for their good knowledge of COVID-19. Likewise, in Germany, perceived vulnerability to COVID-19 infection was associated with adaptation to preventive behaviours [35].

Our results also show that good knowledge of COVID-19 was significantly associated with those who were not misinformed of COVID-19 preventive measures compared to their counterparts. Our finding concurs with a study performed in Saudi Arabia that reported misinformation among the participants [36]. Also in Iran, an association was reported between news media and knowledge of COVID-19 [37]. Media has contributed to information broadcast as a domineering point in decreasing the spread of the COVID-19 virus. However, misinformation could be a hurdle to taking proper preventive measures and optimistic actions toward COVID-19. Hence, the assessment of correct knowledge about a disease is necessary for prevention.

This study showed that those who had good levels of knowledge of effective prevention measures were more likely to have an overall good knowledge of COVID-19. A possible reason is that effective measures are part of the COVID-19 knowledge provided to nations to educate citizens on the COVID-19 virus and its mode of transmission, prevention and control. Our participants who perceived the spread of COVID-19 to be slow were less likely to have good knowledge of the COVID-19 virus than those who perceived it as moderate. This might lead to a tendency where there is a reluctance to seek information since they perceive the transmission rate of the COVID-19 virus to be slow. It is also possible that these respondents reside in areas where the spread of the COVID-19 virus is low and therefore are less likely to be concerned about accurate knowledge.

## Strengths and limitations

The study is a nationwide representative, covering Ghanaians in all 260 districts and 16 regions of Ghana. The study was carried out in collaboration with the health promotion division of the Ghana Health Service, an avenue for health workers to have access to citizens and educate them on the COVID-19 pandemic after participants have finished answering the questionnaire and directing them to the Ghana Health Service website for credible information on the COVID-19 pandemic. Despite the strengths, there are some limitations to the study. First, the study population may not be representative of all Ghanaians due to the small proportion of participants who took part in the study. Second, the study recorded more representatives from tertiary education levels than ordinary Ghanaian citizens because of the data collection method used, which can be accessible to those who have devices with internet connectivity and those who can read and understand. A comparative analysis was constrained by the absence of a variable to distinguish between those who completed the questionnaire independently and those who were contacted by phone.

## Conclusion

The study found good knowledge regarding COVID-19, control measures, preventive strategies and identification of the most vulnerable groups of COVID-19 infections among the respondents. Factors associated with COVID-19 knowledge were age, educational level, region, being free of infection with the novel coronavirus, knowing someone infected, perceived spreading speed, level of knowledge on effective prevention measures and misinformation. We recommend that the Ghana Health Service and the Ministry of Health continuously provide accurate information to educate the media and citizens to prevent misinformation, which is vital in stopping the spread of the COVID-19 virus. Practical educational programs should be provided by public health experts by highlighting the persistent compliance of protective measures for everyone to increase their knowledge of COVID-19.

## Supporting information

S1 File (DO)

S2 File (DO)

S3 File (DTA)

S4 File (XLSX)

S1 Table (XLSX)
